# Motor Cortex Inputs at the Optimum Phase of Beta Cortical Oscillations Undergo More Rapid and Less Variable Corticospinal Propagation

**DOI:** 10.1523/JNEUROSCI.1953-19.2019

**Published:** 2020-01-08

**Authors:** Flavie Torrecillos, Emma Falato, Alek Pogosyan, Timothy West, Vincenzo Di Lazzaro, Peter Brown

**Affiliations:** ^1^Medical Research Council Brain Network Dynamics Unit at the University of Oxford, OX1 3TH Oxford, United Kingdom,; ^2^Nuffield Department of Clinical Neurosciences, John Radcliffe Hospital, University of Oxford, OX3 9DU Oxford, United Kingdom, and; ^3^Unit of Neurology, Neurophysiology, Neurobiology, Department of Medicine, Università Campus Bio-Medico di Roma, 00128 Rome, Italy

**Keywords:** beta oscillations, communication through coherence, corticospinal system, motor cortex, transcranial magnetic stimulation

## Abstract

Brain oscillations involve rhythmic fluctuations of neuronal excitability and may play a crucial role in neural communication. The human corticomuscular system is characterized by beta activity and is readily probed by transcranial magnetic stimulation (TMS). TMS inputs arriving at the excitable phase of beta oscillations in the motor cortex are known to lead to muscle responses of greater amplitude. Here we explore two other possible manifestations of rhythmic excitability in the beta band; windows of reduced response variability and shortened latency. We delivered single-pulse TMS to the motor cortex of healthy human volunteers (10 females and 7 males) during electroencephalography recordings made at rest. TMS delivered at a particular phase of the beta oscillation benefited from not only stronger, but also less variable and more rapid transmission, as evidenced by the greater amplitude, lower coefficient of variation, and shorter latency of motor evoked potentials. Thus, inputs aligned to the optimal phase of the beta EEG in the motor cortex enjoy transmission amplitude gain, but may also benefit from less variability and shortened latencies at subsequent synapses. Neuronal phase may therefore impact corticospinal communication.

**SIGNIFICANCE STATEMENT** Brain oscillations involve rhythmic fluctuations of neuronal excitability. Therefore, motor responses to transcranial magnetic stimulation are larger when a cortical input arrives at a particular phase of the beta activity in the motor cortex. Here, we demonstrate that inputs to corticospinal neurons which coincide with windows of higher excitability also benefit from more rapid and less variable corticospinal transmission. This shortening of latency and increased reproducibility may confer additional advantage to inputs at specific phases. Moreover, these benefits are conserved despite appreciable corticospinal conduction delays.

## Introduction

Oscillations are a ubiquitous phenomenon in the brain and the rhythmic fluctuations in neuronal excitability that they entail may impact on neural communication (Buzsáki and Draguhn, 2004). The motor cortex is one area in the brain where these effects can readily be explored in the human by noninvasively recording electroencephalographic activity (EEG) and probing excitability with transcranial magnetic stimulation (TMS). This brain area has two principal oscillatory modes of synchronized activity which peak in the alpha (also termed mu) and beta frequency bands (Pfurtscheller et al., 1997). Do these oscillations shape neuronal output in the human motor cortex and if so how? Oscillations in neuronal networks help promote synchronization between spikes and hence postsynaptic efficacy through the provision of rhythmic windows of increased neuronal depolarization and excitability (Fries, 2005, 2015). Perhaps the most obvious impact of oscillations in the motor cortex should then be on output amplitude, as indexed by the muscle evoked potential (MEP) in response to TMS. This should vary in tandem with the phase of each wave of the alpha or beta oscillation at the point of stimulation. The evidence for this in the alpha band is mixed (Mitchell et al., 2007; Keil et al., 2014; Berger et al., 2014; Schulz et al., 2014; Iscan et al., 2016; Madsen et al., 2019; Schaworonkow et al., 2019), suggesting perhaps that alpha activity recorded over the motor cortex does not involve major entrainment of the pyramidal neurons projecting to the spinal cord. In contrast, beta activity in the motor cortex does involve pyramidal neurons, as evidenced by corticomuscular coherence (Conway et al., 1995; Kristeva et al., 2007), and TMS inputs locked to the excitable phase of beta oscillations in the contralateral motor cortex or to specific phases of coherent oscillations in the electromyographic activity lead to motor evoked potentials of greater amplitude (van Elswijk et al., 2010; Keil et al., 2014; Khademi et al., 2018).

But is an increased strength of transmission the only advantage conferred upon inputs arriving at the excitable phase of cortical beta oscillations? At least in theory such inputs might also benefit from a shorter delay to discharge of already depolarized (but subthreshold) postsynaptic neurons. And as relative depolarization is synchronized across the neural population, this might also result in a more consistent output amplitude across trials. Evidence for both these effects exists for visually induced gamma band oscillations in cat and nonhuman primate visual cortical areas (Fries et al., 2001; Womelsdorf et al., 2007; Womelsdorf et al., 2012; Besserve et al., 2015; Ni et al., 2016; Rohenkohl et al., 2018). Here we test whether similar effects favoring transmission through shorter latency and greater reproducibility can be seen with regard to beta oscillations in the human motor system.

## Materials and Methods

### 

#### Participants

Seventeen healthy volunteers gave their written informed consent to participate in the study (10 females, age range 19–58 years, mean age 35.3 ± 13 years). The study was approved by the local Research Ethics Committee (Med IDREC Ref: R55269/RE001) and performed in accordance with the Declaration of Helsinki. None of the participants had any contraindications to transcranial magnetic stimulation (TMS) or history of neurological illness. There was strict adherence to the international safety guidelines for TMS (Rossi et al., 2009). All participants were right-handed according to the Edinburgh Handedness Inventory (Oldfield, 1971). The study consisted of two experiments performed in two separate sessions combining EEG recordings and TMS at rest. Fifteen volunteers participated in the first session and seven in the second session recorded 10 months later. Five participants participated in both sessions. The second session was specifically designed based on the results of the first session, to confirm and further characterize our results with a larger number of trials, a better time resolution and an optimized experimental protocol (see Results).

#### Data acquisition

##### Session 1.

**Figure 1.**
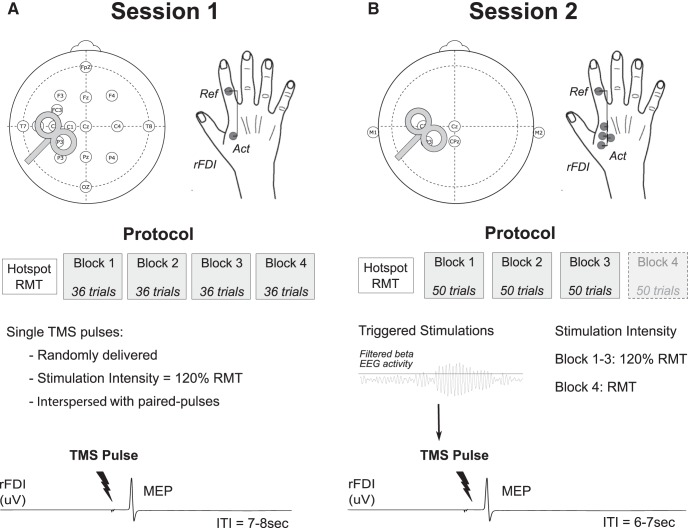
Setup and protocol. ***A***, In the first session, EEG was recorded from 19 electrodes and EMG from the rFDI. Single TMS pulses were randomly triggered with an ITI of at least 7 s. ***B***, In the second session, EEG was recorded from four electrodes and referenced to the average of the two mastoids (M1 and M2). EMG was recorded from the rFDI via four electrodes all referenced to the same electrode (Ref. electrode). Single TMS pulses were triggered by high beta activity and delivered at an intensity of 120% RMT in the three first blocks. For three participants, one block was added with single pulses delivered at RMT intensity.

EEG signals were recorded through an EEG cap from 19 electrodes placed on a subset of the 10/20 system with an increased resolution over the region of the primary motor cortex (C3 electrode; see Fig. 1*A*). The ground Ag/AgCl electrode was placed on the left forearm. EEG data were amplified, acquired at a sampling rate of 2048 Hz and common referenced using a 32-channel TMSi-Porti amplifier and its respective software (TMS International). The same amplifier was used to record surface electromyographic (EMG) activity through Ag/AgCl electrodes placed bipolarly on the muscle belly of the right first dorsal interosseus (rFDI) and the first phalanx of the index finger. EMG signals were sampled at 2048 Hz and bandpass filtered between 8 and 375 Hz.

##### Session 2.

EEG and EMG signals were acquired at a high sampling rate of 20 kHz using a D360 amplifier (Digitimer) in combination with a 1401 A/D converter (Cambridge Electronic Design). The ground Ag/AgCl electrode was placed on the left forearm. EEG signals were recoded from Cz, C3, CP3, and CPz referenced to the average of the two mastoids (M1 and M2). EMG signals were recorded with the same amplifier from only the rFDI. Four active electrodes were placed on the muscle belly and referenced to the electrode placed on the first phalanx of the index finger (see Fig. 1*B*). EMG signals were band-pass filtered between 10 Hz and 10 kHz.

#### Paradigm

In both sessions participants were seated with their right hand rested, palm down on a table beside them. All the recordings were made at rest, with participants instructed to fix their gaze on a fixation point. TMS was performed using a MAGSTIM 200 device (Magstim) and a standard figure-of-eight 70 mm coil delivering a monophasic magnetic pulse. The coil was held tangential to the scalp and angled 45° angle from the sagittal midline to elicit a posterolateral-anteromedial current flow (see Fig. 1). The optimal TMS site to elicit MEPs from the rFDI (“hotspot”) was determined over the left primary motor cortex M1 and hotspot location was marked over a swimming cap placed on top of the EEG cap, to ensure constant coil positioning throughout the experiment. The resting motor threshold (RMT) intensity was determined according to international guidelines as the stimulator's output able to elicit reproducible MEPs of at least 50 uV peak-to-peak amplitude in at least 5 of 10 consecutive stimulations (Rossini et al., 2015). Single-pulse stimulation was then set at an intensity of 120% RMT for all but one block.

The first session consisted of four blocks, in which single pulse stimulations were applied in a random order with an intertrial interval (ITI) between 7 and 8 s to give a total of 36 trials. Note that only 12 single pulse stimulations were delivered in each block as paired-pulse stimulations, not analyzed in the present study, were interspersed in the remaining trials.

The second session consisted of three blocks of 50 single-pulse stimulations specifically delivered at a time of high beta power with an intensity of 120% RMT. To this end, the EEG activity of one preselected electrode (see below) was filtered around the beta peak, rectified and smoothed online using a moving average filter of 200 ms. After 2 min of rest recordings, a threshold was defined to trigger the stimulation with this threshold corresponding to ∼75^th^ percentile of the signal amplitude. An ITI of 7 ± 1.5 s was set before the start of a new screening window. For three participants of the second session, additional single-pulse stimulations were delivered at RMT in a fourth block of 50 trials (see Fig. 1*B*).

#### EMG and EEG preprocessing

##### EMG preprocessing.

MEPs were recorded from the rFDI. For the second session the EMG electrode with the largest averaged MEP amplitude was selected for analysis. Each trial was visually inspected and those showing pre-TMS EMG activation were rejected. Peak-to-peak MEP amplitudes and onset latencies were measured in a semiautomatic manner by using a customized script in Spike2 (version 7.12b; Cambridge Electronic Design). MEP latencies corresponded to the time point at which rectified EMG signals exceeded an amplitude threshold defined as the average 100 ms pre-stimulus EMG activity across all trials plus two SDs (Hamada et al., 2013).

##### EEG preprocessing.

All EEG data preprocessing was performed offline using MATLAB (The MathWorks) and the open-source Fieldtrip toolbox (Oostenveld et al., 2011). EEG recordings were one-pass filtered between 1 and 100 Hz with a forward filter only, to avoid any contamination of the pre-TMS window by the TMS pulse or the post-TMS window. Artifact trials were rejected based on visual inspection. One EEG electrode was selected for each participant based on the reactivity of beta oscillations to movement determined in a short session preceding the EEG-TMS recordings (not analyzed here) in which they performed a cued isometric force production task with their right index finger. The electrode with the largest average movement-related power change in the whole beta band (13–35 Hz), i.e., the largest difference between the trough of the event-related desynchronization (ERD) during movement and the peak postmovement synchronization (ERS), was then selected for further analysis. The selection included C3, CP3, C1 and C5 in 9, 6, 1 and 1 participants respectively. Note that for the five participants performing the two sessions, the same electrode was kept for both sessions. After both EEG and EMG artifact rejection, on average 44.5 ± 1.2 trials for each participant were included in session 1, 121 ± 13 in session 2 (blocks 1–3), and 42 ± 2.5 in the additional RMT block in session 2.

#### EEG phase estimation

The phase of the pre-TMS EEG signal was estimated at the selected electrode (see above) for all frequencies between 5 and 75 Hz in steps of 1 Hz following a similar approach as van Elswijk et al. (2010). For each frequency, an epoch with a length of 2 cycles ending prior the TMS pulse was defined. The epochs were then multiplied by a Hanning taper and Fourier transformed to determine the phase at the respective frequency, which resulted in 70 phase estimations for each single trial. As our subjects were at rest, we were unable to define the pre-TMS phase in the EMG, as performed by van Elswijk et al. (2010).

#### Relationship between pre-TMS EEG beta phase and MEP parameters

For each EEG frequency separately, trials were binned according to their pre-TMS EEG phase. To this end, seven (or nine for the session 2) overlapping bins with equally spaced centers were defined from −π to π. Each trial with a phase included between the bin edges was assigned to that bin. Note that, a single trial may be assigned to more than one bin due to the overlap between them. Single trial MEP amplitudes and latencies were normalized for each participant (*z*-score) before the binning procedure and then averaged across trials for each bin. The strength of the MEP phase-dependent modulation was quantified at each frequency by estimating the difference between the minimum and maximum of a fitted sine function (see below). The average strength of modulation across participants was then statistically compared with a surrogate obtained by shuffling the strength of modulation across frequencies 2000 times for each participant. Cluster-based permutation tests were used to correct for multiple comparisons.

**Figure 2.**
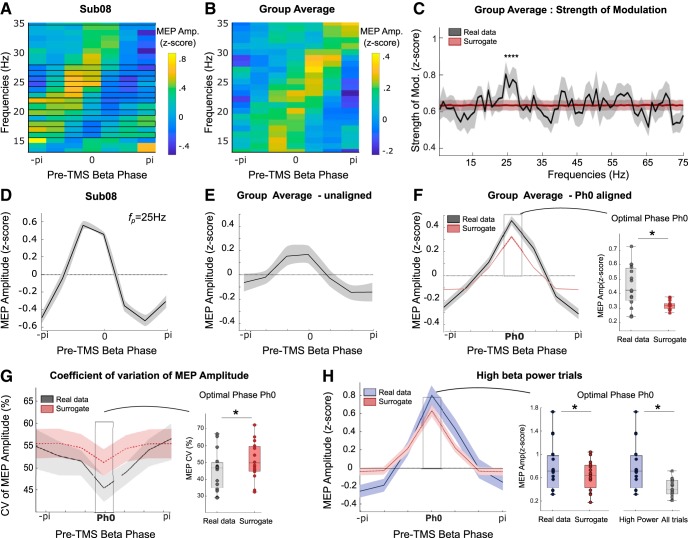
Phase-dependent modulation of MEP amplitude. ***A***, ***B***, MEP amplitude as a function of the pre-TMS EEG phase across the beta band in one participant (***A***) and the group (***B***). Frequencies showing a significant circular-linear correlation between MEP amplitude and pre-TMS beta phase are encased in black. ***C***, Strength of MEP amplitude modulation across participants for all frequencies from 5 to 75 Hz. Cluster-based permutation test revealed a higher modulation of MEP amplitude from 25 to 28 Hz (denoted by asterisk) compared with surrogate. ***D***, Phase-dependent modulation of MEP amplitude for the same participant as in ***A*** in a 3 Hz frequency band centered around fp. ***E***, Phase-dependent modulation of MEP amplitude averaged across all participants. ***F***, Average of MEP amplitude modulation aligned to each respective optimal phase Ph0 and compared with a surrogate (dashed). Paired *t* tests at Ph0: *p < 0.05. ***G***, CV of MEP amplitude across phase bins when the optimal phases of each participant are aligned. Repeated-measures ANOVA (*F*_(6,14)_ = 3.98, *p* = 0.0015). Paired *t* test for comparison with surrogate: *p < 0.05. ***H***, Phase-dependent modulation of MEP amplitude when beta pre-TMS beta power is high (median split of all trials) compared with surrogate data or the modulation previously observed at Ph0 when all trials were considered. Paired *t* tests at Ph0, *p < 0.05.

Group data suggested phase-dependency was confined to the beta band (see Fig. 2*C*) and based on this, and previous findings (van Elswijk et al., 2010; Khademi et al., 2018), detailed examination of the phase-dependent modulation of MEPs was focused on the beta band (from 13 to 35 Hz). To test for a dependency of MEP features on the pre-TMS EEG phase, the following approach was taken. First, circular-linear correlations were tested at each frequency between the normalized MEP amplitude or latency and the pre-TMS EEG beta phase with the *cirr_corrcl* function of the CircStat MATLAB toolbox (Berens, 2009). The sine wave shaped relationship was further tested by fitting a sine function to the phase-dependent modulation at each frequency.

Second, one phase-specific frequency (*f*_p_) was selected for each participant in the beta band. The individual *f*_p_ was defined as the frequency with the largest MEP amplitude modulation, i.e., the largest difference between the minimum and maximum of the fitted sine function. Based on the observation that phase dependency was also observed at bordering frequency bins to *f*_p_ (supported by the diagonal shift of maximal MEP amplitudes seen in Fig. 2*A*) the modulation at each phase was estimated as the average of the normalized MEP amplitude across a 3 Hz frequency band centered on *f*_p._ To be able to average at a constant time across the three 1 Hz bins comprising the 3 Hz band, modulations were normalized to the center *f*_p_ frequency, by correcting the phase estimates of the upper and lower 1 Hz band for the period difference with the central band. Finally, to average across participants, the phase bins with the largest MEP amplitude were realigned to 0° phase for each participant. In addition, the trial-by-trial variability of MEPs was assessed within each bin by the coefficient of variation (CV) defined as the ratio between the SD of the MEP parameter in the bin and its mean.

For session 1, phase modulations were further investigated in trials with elevated pre-TMS beta power. To this end trials were median split according to the beta power in a 200 ms window preceding the TMS pulse.

#### Distribution of MEP latencies and components

To look at their distribution, MEP latencies of session 2 were mean corrected for each participant by subtracting the mean MEP latency of all trials from single MEP latencies. The width of the bins was fixed to 0.2 ms for all distributions. The probability densities were estimated based on a normal kernel function and evaluated at equally spaced points. The peaks were defined as the maximum of the probability density estimates.

MEP subcomponents were identified as follows. First EMG responses were epoched into a time-window extending from 5 ms before the MEP onset to the time point when the undifferentiated, raw EMG signal reversed polarity (see Fig. 6*A*, black line). Thus the duration of epochs varied across trials and participants (averaged duration: 10.1 ms ± 0.3 ms) with a minimum of 8.8 ms ± 0.35 ms. Second, EMG was differentiated and local peaks identified when the differentiated signal exceeded a threshold equivalent to the mean baseline differentiated EMG activity plus 3 SDs. The baseline EMG activity was the averaged differentiated EMG signal recorded from 5 ms after the TMS pulse to 5 ms before the MEP onset (i.e., to the start of the epoched MEP responses). Local peaks had to be separated by a minimum interval of 0.25 ms (5 data points) to be considered separate. Once identified the timing of the local peaks in each trial was realigned to the time of the averaged MEP peak for each participant. The probability distribution of timings was then found for all participants (see Fig. 6*B*) and the optimal Gaussian mixture fit determined by comparing the AIC of the fits for one to five components.

#### Model

We formulated a model of the corticospinal pathway to describe how presynaptic activation of corticospinal cells in response to TMS results in multiple EPSPs that propagate along the corticospinal tract and result in a MEP. The model comprises a three-layer hierarchical spiking network with the architecture illustrated in Figure 7*A*. The three layers correspond to populations of 24 excitatory corticospinal neurons; 20 excitatory alpha motor neurons; and one muscle. We model the spatiotemporal summation of TMS evoked activity in the cortex along the pathway using coupled leaky integrate and fire neurons.

Each cortical neuron produces a train of spikes at times when the membrane voltage exceeds a firing threshold. The firing threshold was set by determining an upper bound on the probability of firing a spike with each TMS impulse and was set at 80% success to match the empirical observations. A refractory period of 2 ms was set to restrict maximum spike rates of individual units (Maier et al., 2013). Target neurons in the layer below were chosen at random such that each corticospinal neuron projected to four target alpha motor neurons; and all alpha motor neurons fed forward to the muscle.

At each receiving neuron, spike trains were spatially summated across the source neurons to form the total presynaptic input. This summated input *s*(*t*), was convolved with a postsynaptic response kernel κ(*t*), to convert from a point process of spikes to a continuous process describing the postsynaptic membrane voltage of the target neuron:

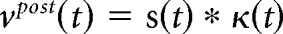
 The postsynaptic kernel describing the EPSP was described by a biexponential function with two-time constants τ_1_ and τ_2_ describing a fast rise and slow decay of the membrane voltage in response to a synaptic input:

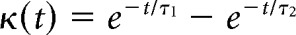
 To simulate noise related variability of the EPSPs, we introduced a jitter to the summated presynaptic spike train such that the amplitude of the resulting EPSPs were normally distributed with a SD of 10% of the mean amplitude. Additionally, the corticospinal neuron population received a synchronized, subthreshold, periodic membrane depolarization at the beta frequency (see Fig. 7). The evolution of this process was first described in the frequency domain using a Lorentzian frequency spectrum with center frequency ω_0_, full width at half maximum γ, and random phase φ:

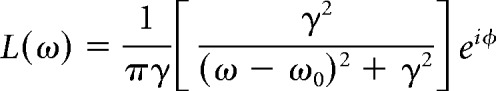
 where the phase is uniformly distributed around the unit circle. We set a center frequency and full width at half maximum at 20 Hz and 0.5, respectively. The background beta depolarization in the time domain is then formed by the inverse Fourier transform:

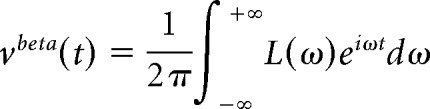
 Finally, the effect of the TMS pulse upon corticospinal neurons was simulated with three EPSPs separated by 2 ms, and modeled as probabilistic events with amplitudes *I*_1,_ *I*_2_, and *I*_3_ chosen such that *I*_1_ > *I*_2_ > *I*_3_, and an associated probability of the cortical neurons being in receipt of the wave chosen such that *p*(*I*_1_) > *p*(*I*_2_) > *p*(*I*_3_). Thus, these EPSPs were modeled such that they might lead to the *I* waves typically recorded experimentally from the cervical cord (Di Lazzaro et al., 2018). The resulting TMS evoked EPSP at the cortical neuron ν^IN^, assumed to arise due to trans-synaptic input from TMS excitable interneurons, was again made by convolving a pair of spikes (*s*^TMS^) with a synaptic kernel (κ^CSN^) for the corticospinal population:

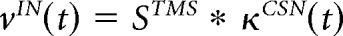
 Thus, the total input to an individual CSN (at the first level of the network) is equal to:

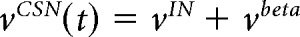
 The input to the individual alpha motor neurons is equal to:

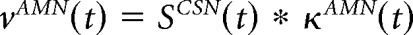
 And finally, the potential evoked at the muscle endplate is given by the following:

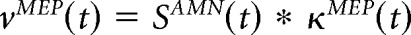


#### Statistics

Statistical analyses were performed using the free software R and custom-written MATLAB routines. Mean *z*-scored MEP amplitudes or latencies at the optimal phase were tested against surrogate data by means of paired *t* tests, after examination of the normality assumption. Surrogate data were obtained by shuffling the original data across phase bins and realigning the peaks of individual shuffled amplitude-phase profiles to zero radians. This was repeated 1000 times before averaging to give mean shuffled data for each subject. These were then averaged across participants. CVs of MEP amplitude were compared with similarly treated surrogate data and also between phase bins with repeated-measures ANOVA. Huyn-Feldt correction was applied whenever appropriate. The overlap of the beta frequencies selected for their phase-modulation of MEP amplitude and latencies (*f*_p_) was compared with a paired signed rank test (Wilcoxon test) to a surrogate obtained by shuffling the *f*_p_ 1000 times. Finally, the distributions of MEP onset latencies at the optimal and nonoptimal phases were compared by a two-sample Kolmogorov–Smirnov test.

The probability distribution of timings for the optimal and nonoptimal phases (see Fig. 6*B*) were compared by testing, at each time point, whether the original difference is higher than what would be observed by chance. To this end, a distribution “by chance” was estimated by shuffling the original data 1000 time. Then, at each time point, a *z* test was applied to test whether the original difference came from the same distribution. Finally, cluster-based permutation tests were used to correct for the multiple comparisons along the time axis.

## Results

### Response size and variability depend on EEG phase in the beta band

Before testing our hypothesis that inputs arriving at the excitable phase of cortical oscillations receive a latency advantage in the corticospinal system, we first confirmed the phase-dependency of MEP amplitude (van Elswijk et al., 2010; Keil et al., 2014; Khademi et al., 2018). To this end, MEPs acquired at rest during single pulse TMS (Fig. 1*A*) were analyzed as follows. First, single-trial MEP amplitudes were normalized (*z*-score) for each participant to the mean MEP amplitude evoked by all single pulse stimulations in that participant. Second, the trials were sorted for each 1 Hz frequency increment in the beta-frequency band from 13 to 35 Hz according to the EEG phase immediately preceding the TMS pulse and MEP amplitudes averaged across trials. Phase was divided into seven equal bins. The variation of MEP amplitude with phase is shown across the beta frequency range as color scaling for an example participant in Figure 2*A*. The diagonal shift of maximal MEP amplitudes with ascending frequency was common across participants, as can be seen from the group average (Fig. 2*B*). This linear shift in phase is due to excitabilities aligning at a constant time lag across frequencies and in of itself suggests a dependency of MEP amplitude on phase across frequencies within individual subjects. The dependency of MEP amplitude on pre-TMS EEG phase was further tested by circular linear correlations at each frequency of the beta range. For each participant at least one frequency with a significant correlation was found in the beta-band, with on average 4.6 ± 0.9 frequencies per participant. Given this relatively high incidence of circular linear correlation, we tested the specificity of the phase-dependent modulation in the beta band by considering frequencies from 5 to 75 Hz. For each participant and frequency the strength of MEP amplitude modulation was quantified by fitting a sine function and taking the difference between the maximum and minimum of the fitted function. The result was compared with a surrogate distribution obtained by shuffling the data across frequencies 2000 times for each participant (Fig. 2*C*). A cluster-based permutation test demonstrated that the MEP amplitude of the original data was significantly higher in the beta-band frequency range, from 25 to 28 Hz. Based on these results only the beta frequency range was considered for further analysis.

The peak phase-modulated beta frequency (*f*_p_) was thereafter defined as the beta frequency with the highest strength of modulation and the modulation at each phase was estimated from the average of the normalized MEP amplitude across a 3 Hz frequency band centered on *f*_p_ in each participant (Fig. 2*D*, same participant as Fig. 2*A*). The average *f*_p_ was 25 ± 2 Hz across participants. Note that although similar patterns of phase dependency were observed for all participants with a sine wave shaped relation and only one peak of excitability per cycle, peaks could occur at different beta phases among participants due to the individualized selection of *f*_p_. Thus, as the phase of peak modulation depended on *f*_p_, only weak modulation was observed at the group level (Fig. 2*E*). To circumvent this confound, the peaks of individual amplitude-phase profiles in subject averaged data were realigned to zero radians before averaging across participants (Ph0; Fig. 2*F*, black line). Such a realignment would create artificially a positive modulation at the optimal phase, even for data that are pure noise. Thus, MEP amplitudes were tested against surrogate data (data shuffled 1000 times, see methods) that includes the modulation created by the peak realignment procedure. Statistical results revealed a significantly stronger modulation of MEP amplitude at the optimal phase Ph0 (*t*_(14)_ = 3.33, *p* = 0.005). This result is consistent with the communication by coherence theory, in which postsynaptic effects are thought to be greater when an input arrives at the phase in the oscillation of the target neuronal population that corresponds to a maximum depolarization. Notably, not only was this postsynaptic effect greater at this phase it was also less variable from trial to trial. Thus a significant modulation of the CV of MEP amplitude was found across phase bins (*F*_(6,14)_ = 3.98, *p* = 0.007) with a minimum at the optimal Ph0 phase (Fig. 2*G*, comparison to surrogate *t*_(14)_ = 2.38, *p* = 0.03). This modulation of CV with phase illustrates that MEP amplitudes are less variable from trial to trial when the TMS pulse is delivered at the optimal phase, even when allowing for the phase realignment of individual amplitude peaks and provides further evidence that such realignment is physiologically meaningful. Finally, the phase-dependency of MEP amplitude by cortical beta phase was increased when trials were median split by pre-TMS EEG beta power and the higher-power trials considered (Fig. 2*H*, paired *t* test high EEG beta power trials against surrogates *t*_(14)_ = 2.24 *p* = 0.04 and against all trials, *t*_(14)_ = 2.79 *p* = 0.04).

### MEP latency depends on EEG phase in the beta band

**Figure 3.**
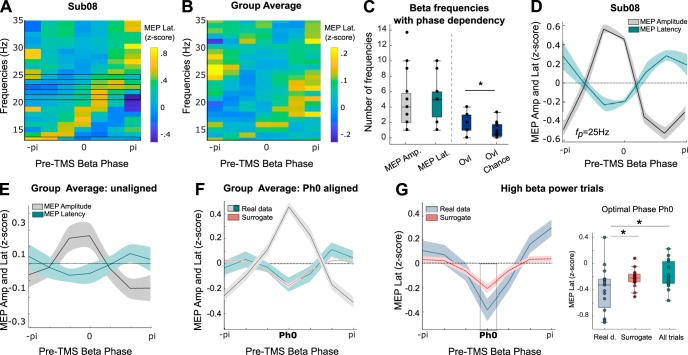
Phase-dependent modulation of MEP latency. ***A***, ***B***, MEP latency as a function of the pre-TMS EEG phase across beta frequencies in one participant (***A***) and the group (***B***). Frequencies showing a significant circular-linear correlation between MEP latency and pre-TMS phase are encased in black. ***C***, Number of beta frequencies showing a significant phase-dependent modulation of MEP amplitude or latency (*f*_p_) for all participants and number of fp showing a significant modulation of both (Ovl). The overlap of fp (Ovl) is higher than by chance (Ovl Chance, *p < 0.05, signed-rank test). ***D***, Phase-dependent modulation of MEP amplitude and latency for the same participant as in ***A*** in the 3 Hz frequency band. ***E***, Phase-dependent modulation of MEP amplitude and latency averaged across all the participants. ***F***, Phase dependency of MEP latency when peaks of MEP amplitude modulations are realigned to zero (Ph0). The MEP amplitude modulation is shown for convenience (same as in Fig. 2*F*). ***G***, Phase dependency of MEP latency aligned to Ph0 when only high pre-TMS beta power trials are considered. Paired *t* tests at Ph0 against surrogate or all trials: *p < 0.05.

Having established that a single TMS pulse at the optimal phase of beta cortical activity leads to a stronger and more consistent corticospinal output we proceeded to test our core hypothesis that inputs at the optimal phase for amplitude modulation will also lead to postsynaptic activations at shorter latency. In a later section we exclude changes in MEP amplitude as an explanation for changes in MEP latency in our paradigm, but we begin by reporting the main findings. The procedures described above for MEP amplitudes were repeated for MEP latencies and revealed a similar pattern with a diagonal shift of MEP latencies with ascending frequency (see Fig. 3*A* for same subject as Fig. 2*A*; see Fig. 3*B* for group average data). This suggests a dependency of MEP latency on phase across frequencies within individual subjects. In each participant, at least one beta frequency showed a significant circular-linear correlation between MEP latencies and the pre-TMS EEG phase indicating phase-dependency (on average 4.9 ± 0.6 frequencies per participant; Fig. 3*C*). For each participant, the modulations of MEP amplitude and latency tended to occur at overlapping beta frequencies more than predicted by chance (*f*_p_ shuffled 1000 times, paired sign-rank test, *W* = −86, *p* = 0.012; Fig. 3*C*). For further analyses only the beta frequency with the highest MEP amplitude modulation was considered (*f*_p_) and latency modulation was analyzed following the exact same procedure as for MEP amplitude.

Figure 3*D* shows MEP latency as a function of the beta phase at which the TMS pulse was applied for the same participant as shown in Figures 2, *A* and *B*, and 3*A*. The modulation of MEP amplitudes is also shown again for convenience. Both amplitude and latency present a sinusoidal profile with pre-TMS EEG phase predominantly in antiphase. The same pattern was evident in the unaligned group average although not significant (Fig. 3*E*). As before, the peaks of MEP amplitude modulations were realigned to zero (Ph0), and the same phase shifts applied to the corresponding latencies, before averaging. In contrast to the MEP amplitude, no significant modulation of MEP latency was observed at the optimal phase when compared with surrogate data (*t*_(14)_ = −0.5, *p* = 0.6; Fig. 3*F*) and no significant effect was observed for the variability of MEP latency (*F*_(6,14)_ = 1.12, *p* = 0.36). The latter might be explained by the fact that MEP latencies are typically more stable within individuals than MEP amplitudes (Kiers et al., 1993). In line with this, the average CV of MEP amplitudes was 54 ± 3% across participants compared with 8 ± 6% for MEP latencies. Motivated by the previous findings, the MEP latency modulation was also studied in trials with elevated EEG beta power immediately before TMS. As illustrated in Figure 3*G*, this lead to an antiphase modulation of MEP features that is even more marked than when all trials were considered together (*t*_(14)_ = −2.8, *p* = 0.014), with, importantly, a significant shortening in latency at Ph0 when compared with surrogate data (*t*_(14)_ = −2.73, *p* = 0.016). These results confirmed the antiphase relation between the two MEP parameters, with the phase associated with the highest increase in MEP amplitude also associated with significantly shorter latency MEPs across participants when considering median split trials with high EEG beta power. The results also highlight the importance of the pre-TMS beta power, which when elevated allows a more dependable estimation of the EEG phase and suggests greater oscillatory synchronization at the cortical level.

**Figure 4.**
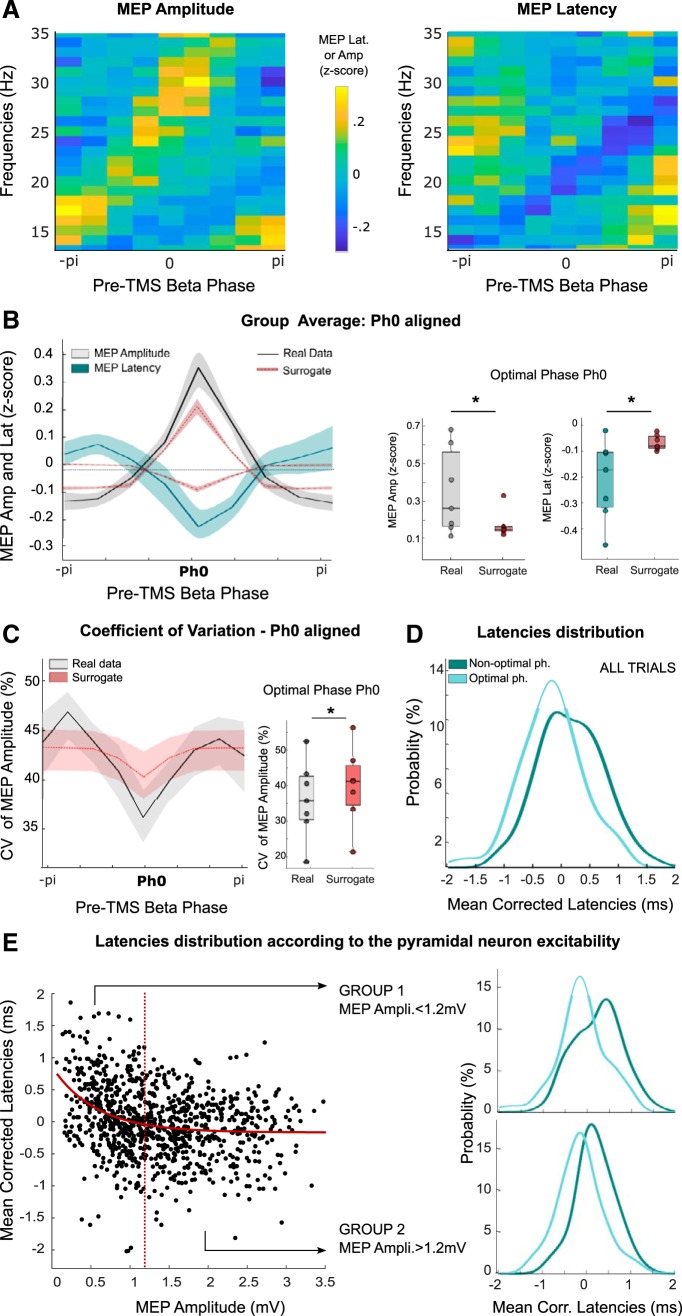
TMS when EEG beta power is high. ***A***, MEP Amplitude as a function of the pre-TMS EEG phase across beta frequencies averaged across all participants. ***B***, MEP Amplitude and latency as a function of the pre-TMS EEG phase averaged across all participants, aligned to the respective optimal phase Ph0 for MEP amplitude and compared with surrogate data. Paired *t* tests at Ph0: *p < 0.05. ***C***, CV of MEP amplitude across phase bins when the optimal phases of each participant are aligned as in B. Repeated-measures ANOVA (*F*_(8,6)_ = 3.25, *p* = 0.005). Paired *t* test against surrogate at Ph0: *p < 0.05. ***D***, Distribution of mean-corrected MEP latencies of all participants at their respective optimal and nonoptimal phases for all trials (probability density function). ***E***, Mean-corrected MEP latencies according to the pyramidal neuron excitability as inferred from MEP amplitude with fixed stimulation intensity. Two groups of trials were defined based on a 1.2 mV threshold (dashed red line) and the distribution of mean-corrected MEP latencies of all participants at their respective optimal and nonoptimal phases were compared for Group 1 (trials with MEPs < 1.2 mV) and Group 2 (MEPs > 1.2 mV).

To corroborate and further characterize the phase dependency of MEP latency we ran a second experiment. This had a larger number of trials and a higher temporal resolution, and we now specifically delivered single TMS pulses at a time of high beta power to optimize phase-dependency (Fig. 1*B*). The analysis was the same as above, except that the larger number of trials allowed us to increase the resolution of phase estimates by using nine phase bins instead of seven. As before, there was a diagonal shift of maximal MEP amplitudes and latencies with ascending frequency common to all participants (Fig. 4*A*). Again, there was a significant increase in MEP amplitude at the optimal phase, Ph0, accompanied by a significant decrease in MEP latency compared with surrogate data (Fig. 4*B*, *t*_(6)_ = 2.5, *p* = 0.04, and *t*_(6)_ = −2.7, *p* = 0.036, respectively). The phase-dependent reduction in CV was also confirmed for MEP amplitude, where there was less variation across trials at Ph0 compared with surrogate data (Fig. 4*C*, *t*_(6)_ = −5.9, *p* = 0.001). As before, a phase-dependent improvement in CV was only observed for MEP amplitude, with the average CV across all phases of 42 ± 3% for MEP amplitude, as opposed to only 3.2 ± 0.5% for latency.

To better visualize and quantify the shortening of latency at the optimal phase we contrasted the distributions of MEP latencies in the two extreme phase bins; the optimal phase bin, with the shortest latencies, and the bin with the longest latencies (nonoptimal phase). So as to enable comparison across participants MEP latencies were mean corrected. As can be seen in Figure 4*D*, the group distribution of MEP latencies is shifted to the left at the optimal phase (negative values, shorter latency) and to the right at the nonoptimal phase (Kolmogorov–Smirnov test, *k*_stat_ = 0.3, *p* < 0.001). The individual distributions revealed a consistent and robust reduction of latencies in optimal phase trials with differences ranging from 0.2 to 0.7 ms across participants (mean 0.4 ms, *Z* = 28, *p* = 0.016). Further analysis revealed that the shortening of latency was also dependent on MEP amplitude across subjects (Fig. 4*E*). In particular, trials with a MEP amplitude <1.2 mV could be of either relatively long or short latency, whereas those with a MEP amplitude of >1.2 mV were more likely to be of relatively short latency. Note that the cutoff value of 1.2 mV was selected as it afforded a sufficient number of trials in each group (on average 65 ± 13 trials and 69 ± 13 trials) for all the participants. Among trials with a lower MEP amplitude those in which TMS was delivered at the optimal phase tended to have shorter latencies than those in which TMS was delivered at the nonoptimal phase. The latency probability plot contrasting these two subgroups of trials demonstrated two peaks separated by ∼0.7 ms (Fig. 4*E*). The individual distributions revealed a consistent and robust reduction in latencies in optimal phase trials with differences ranging from 0.2 to 0.9 ms across participants (mean difference of 0.5 ms; *Z* = 28, *p* = 0.016).

**Figure 5.**
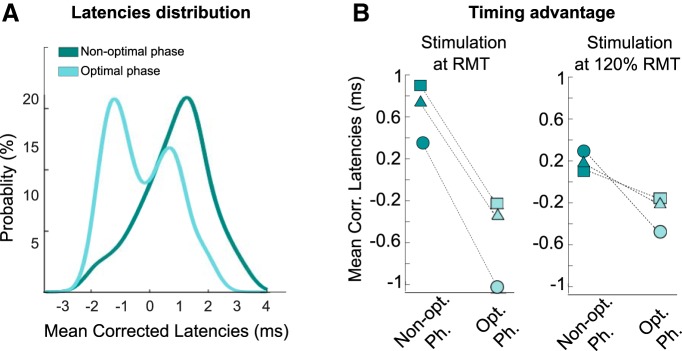
RMT intensity TMS blocks. ***A***, Distribution of mean-corrected MEP latencies at their respective optimal and nonoptimal phases (probability density function). Data from three participants who underwent an additional experimental block with TMS pulses applied at resting motor threshold. ***B***, Mean latencies at the optimal and nonoptimal phases for each of the three participants when TMS pulses were applied at 120% RMT and, in the same subjects, when TMS pulses were applied at RMT.

The split of trials into two subgroups suggested a greater latency shortening in the small MEP amplitude group (Group 1 in Fig. 4*E*) compared with the large MEP group (Group 2). We explored this further by looking at the phase dependency of MEPs elicited by TMS pulses applied at resting motor threshold (RMT). To this end, we added one block of 50 trials for the three last participants of the second experiment. After confirming the phase dependency of both the MEP amplitude and latency we quantified the shortening of latency for each participant in these blocks of TMS at RMT. The results revealed that the MEP latencies elicited by TMS at optimal and nonoptimal phases differed by at least 1 ms in each participant resulting in two well separated distributions at the group level (Fig. 5*A*). Thus the shortening of MEP latency at optimal phase was most marked when TMS stimulation was applied at low intensity, eliciting very small MEPs. This is highlighted in Figure 5*B* where the change in latency according to phase with stimulation at RMT is contrasted with the change in latency according to phase with stimulation at an intensity 20% above RMT in the same subjects. At low stimulation intensities we propose that short latency MEPs reflected EMG responses to the very earliest *I* waves which could only occur when the phase of the cortical beta activity meant that corticospinal neuron excitability was at its greatest. Outside of this phase alpha motor neurons were less excitable and only fired in response to the *I*_2_ wave or even the *I*_3_ wave. With stimulation at higher intensity (Figs. 4*E*, 5*B*) the phase of cortical beta activity conferred less timing advantage as the excitability of the corticospinal neuron was less critical in the face of a large cortical input.

### Dependency of MEP latency on EEG phase is not simply a product of changes in MEP amplitude

Finally, we ruled out an important confound, that a steeper MEP amplitude rise time might account for the difference in latency in trials where TMS was delivered at the optimal phase and nonoptimal phase, considering that the former trials were also associated with larger MEP amplitudes. Larger MEPs may have steeper rising slopes than smaller MEPs, so that the threshold used to define MEP latency might be crossed earlier. The difference in latency would then only be the result of the difference in MEP amplitude. Thus, an important control was to establish whether rising slopes were similar between the two groups of MEPs. First, we determined the MEP slope at the point at which EMG crossed the threshold used for determining MEP latency in sessions 1 and 2. These slopes did not differ significantly between the MEPs at the optimal and nonoptimal phases (paired sign-rank tests; session 1, *W* = 30, *p* = 0.095, session 2, *W* = 8, *p* = 0.38). Similarly, there was no correlation between the latency difference and the difference in MEP slopes between the trial types (session 1, *r* = 0.1, *p* = 0.72, session 2, *r* = 0.5, *p* = 0.25).

**Figure 6.**
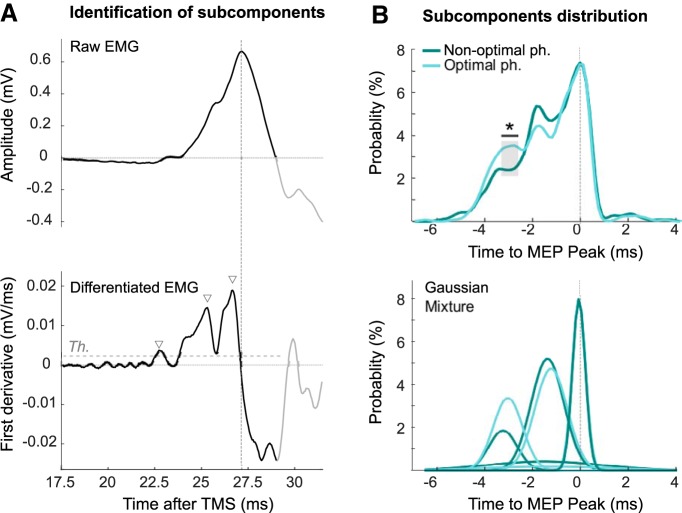
Distribution of MEP components between discrete windows of preferred timing. ***A***, Description of the methods used to identify the different subcomponents. EMG signals from one trial of one participant. EMG derivative was thresholded from 5 ms before the onset of the MEP to the end of the positive (up-going) phase of the EMG amplitude trace (black line). The threshold to be exceeded was equivalent to mean baseline EMG activity plus 3 SDs (see Materials and Methods). The identified subcomponents are marked by the triangles. ***B***, Top, Distribution of subcomponent timings with respect to the timing of the subjects' peak MEP amplitude, for both the optimal and nonoptimal phase. *Difference between the two distributions higher than by chance. Bottom, The two distributions are best fitted by a mixture of four Gaussians. One Gaussian was very small and broad. The remaining three Gaussians corresponded to the peaks in the group data and had timings compatible with a series of *I* waves.

Second, we sought further evidence that the difference in MEP onset latency between stimulation at optimal and nonoptimal phases of cortical beta was due to a change in the distribution of MEP components between discrete windows of preferred timing, rather than due to a continuous shift in latencies. The latter would be the case if changes in latency were just due to changes in the timing of amplitude threshold crossing due to MEP size. The former would suggest that latency changes in MEPs might support our hypothesis that latency changes were due to responses to *I* waves with preferred timings. The similarity between the response to *I* waves seen in poststimulus time histograms of single motor units and the latency and polyphasic nature of muscle responses in the first dorsal interosseous muscle was noted soon after the introduction of transcranial magnetic stimulation (Day et al., 1989). Accordingly, we sought evidence that MEP components occurred within relatively discrete time windows, and that the shift to an earlier time window might account for shortening of latency. To this end, we differentiated MEP responses to emphasize EMG subcomponents and thresholded the resulting signal to give a histogram of the preferred timings of these events with respect to the timing of the subjects' peak MEP amplitude (by way of normalizing latencies across subjects; Fig. 6*A*). Probability density functions and their fit to a Gaussian mixture model revealed a series of three relatively discrete peaks across the subjects (Fig. 6*B*), that have timings compatible with a series of *I* waves (Di Lazzaro et al., 2018). Critically, they also revealed a shift to favor earlier timings in optimal phase trials that differed from what would be obtained by chance (*z* test with cluster-based permutation correction, one significant cluster from −3.5 to −3 ms before the MEP peak, Z = 16.11, *p* < 0.001; Fig. 6*B*).

These findings help exclude the possibility that latency shortening is a mere consequence of increased MEP size, as amplitude was effectively disregarded in the thresholding process used to derive the histograms.

### Modeling EEG phase-dependent effects on MEPs

**Figure 7.**
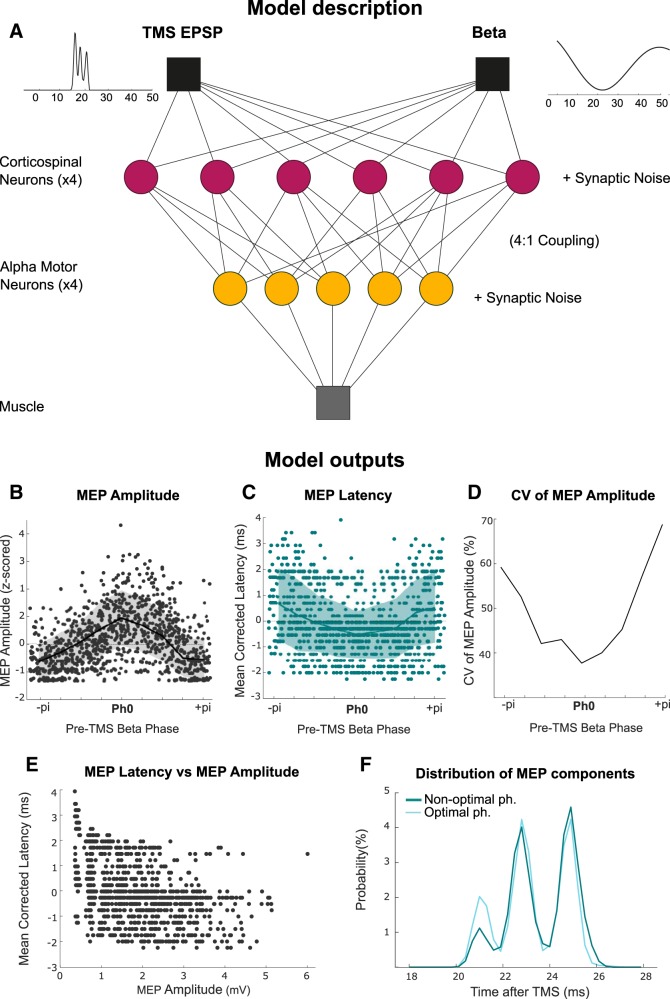
Model of the corticospinal tract capturing the phase dependency of MEPs. ***A***, Description of the model which comprises a three-layer hierarchical spiking network with 24 excitatory corticospinal neurons, 20 excitatory alpha motor neurons and one muscle. The response of corticospinal cells to TMS pulse evoked presynaptic activity was simulated with three EPSPs separated by 2 ms and with higher amplitude and probability for the first EPSP (*I*_1_ > *I*_2_ > *I*_3_) to reflect the nature of subsequent *I*_1_, *I*_2_, and *I*_3_ waves (TMS EPSP, left). The corticospinal neurons also received a subthreshold membrane depolarization at beta frequency (Beta, righ*t*). EPSP: Excitatory postsynaptic potential. ***B***–***F***, Model outputs: Phase dependency of MEP amplitude (***B***), MEP latency (***C***), CV of MEP amplitude (***D***), relationship between mean corrected MEP latency and MEP amplitude (***E***), and distribution of the MEP subcomponents (***F***).

Given the above findings and the fact that MEPs in hand muscles are known to be driven by a high-frequency series of discrete descending volleys in the corticospinal tract (Di Lazzaro et al., 2018) we determined whether the nature and pattern of *I* waves is sufficient to explain the observed differences in MEP behavior according to the phase of cortical beta activity at which TMS is delivered. To this end the corticospinal pathway was modeled by a three-layer hierarchical spiking network as illustrated in Figure 7 (see Materials and Methods for further details). The first layer, corresponding to a population of corticospinal neurons, received both a subthreshold membrane depolarization at beta frequency and up to three EPSPs (excitatory postsynaptic potential). The latter are thought to be induced by the effect of the TMS pulse on cortical interneurons, and ultimately lead to *I*_1_, *I*_2_ and *I*_3_ waves (Di Lazzaro et al., 2018). The corticospinal neurons project on to alpha motor neurons (second layer) and finally to the muscle (layer three) where the amplitude and latency of the response depends on the spatiotemporal summation of TMS evoked activity in more proximal layers. The model explains the phase dependency of the different MEP features (Fig. 7*B–D*), as well as the dependence of the shortening of latency on MEP amplitude (Fig. 7*E*) and the distribution of the discrete EMG peaks (Fig. 7*F*).

## Discussion

Our results show that inputs delivered at a particular phase of cortical beta oscillations benefit from not only stronger but also more rapid and consistent transmission as evidenced by the greater amplitude, shorter latency, and lower CV of motor evoked potentials. We should start by considering two possible confounds. First, a steeper MEP amplitude rise time might account for the difference in latency in trials where TMS was delivered at optimal and nonoptimal phases, considering that the former trials were also associated with larger MEP amplitudes. In that case the link between shortening of latency and increase in MEP amplitude would not be of physiological significance. However, this possibility was discounted by considering MEP slopes at the point of EMG threshold crossing and by demonstrating that the difference in MEP onset latency between stimulation at optimal and nonoptimal phases of cortical beta was due to a change in the distribution of MEP components between discrete windows of preferred timing, rather than due to a continuous shift in latencies. Second, we realigned the phases giving the maximum MEP amplitude across subjects, thus favoring a spurious peak MEP amplitude at phase zero in group data. However, the diagonal shift of maximal MEP amplitudes and latencies with ascending frequency and the circular linear correlations between MEP amplitudes and latencies and phase provided realignment-independent evidence of a dependency of MEP characteristics on phase across frequencies within individual subjects. In addition, phase aligned data were contrasted to similarly treated surrogate data and MEP amplitudes and latencies remained significantly different at zero phase.

We should also consider another possible criticism of our findings. This is that, at least at low MEP sizes, there was a negative correlation between MEP onset latency and MEP peak amplitude (Fig. 4*E*), and therefore our findings with respect to latency might be inevitable given this correlation. We do not dispute this negative correlation, but here rather seek to explain and further characterize it. As discussed below, we ascribe it to shifts between the likelihood of different *I* waves according to cortical excitability. Thus, in our second experiment, MEP latencies and their shifts were better modeled as shifts between different discrete preferred timings rather than by a single Gaussian distribution that shifted in its mean. The former result suggests something more than a simple continuous inverse correlation between MEP onset latency and peak amplitude.

Our core finding was that a significant shortening of MEP latency occurs when TMS inputs to corticospinal neurons are delivered at an optimal phase of beta activity in the EEG activity recorded over the motor cortex. Improvements in the fidelity of information transfer are thought to occur when input arrives at the depolarising, maximally excitable phase of any oscillation in the target neuron (Fries, 2005, 2015). We ascribe the shortening of MEP latency to a similar phenomenon in the corticospinal neuron, as its excitability state alternates with the cortical beta activity. As the EEG is thought to largely reflect the impact of synchronized postsynaptic potentials in pyramidal neurons, like those giving rise to the pyramidal tract, EEG beta activity can be taken as a proxy for oscillatory membrane potential changes of a similar frequency in corticospinal neurons (Buzsáki et al., 2012). We delivered single-pulse TMS with a focal coil over the cortical hotspot for hand muscles with a postero–anterior orientation. At near threshold stimulation intensities this kind of TMS stimulation evokes an *I*_1_ wave, a descending corticospinal volley that is thought to originate from the effect of trans-synaptic input to layer V corticospinal neurones (Di Lazzaro et al., 2018). Later indirect waves (*I*_2_, *I*_3_ etc) can be evoked by higher stimulation intensities, or if the excitability of the corticospinal neuron is raised (Di Lazzaro et al., 1998). Depending on both spatial and temporal summation, these volleys depolarize alpha motor neurons and in turn elicit short-latency MEPs.

Very small decrements in MEP latency at an optimal phase might arise because the layer V corticospinal neurons that are brought to near threshold by the trans-synaptic TMS input, are more excitable and thus are more likely to cross the threshold earlier at this phase of the cortical beta cycle. Without the subthreshold depolarization accompanying beta oscillations corticospinal neurons may need slightly longer to reach discharge threshold as it may take additional depolarization due to noise-related fluctuations in membrane potential to finally bring neurons to threshold, particularly when TMS intensity is low. However, we propose that the main shortening of MEP latency observed in our data is due to the knock-on effects of TMS falling at the phase of the cortical beta oscillation that entails greater subthreshold depolarization of corticospinal neurons. The pool of corticospinal neurons discharging in response to the TMS input will increase at this phase, so that the descending volleys are amplified, and, due to the substantial convergence of corticospinal inputs on to alpha motor neurons (Porter, 1985), spatial summation occurs at the alpha motor neuron. It is the latter that is key and promotes discharge with progressively earlier *I* waves (Di Lazzaro et al., 2018), as descending volleys are amplified. The net effect is a distribution of MEP latencies that has more than one peak, as some trials reflect alpha motor neuron discharge to *I*_1_ or *I*_2_ descending volleys and others to still later volleys. The same mechanism explains the presence of multiple components to MEPs, separated by intervals of just over 1 ms and compatible with the response to successive *I* waves (Day et al., 1989). Phase-dependent shifts in MEP latencies are more pronounced at lower TMS intensities, where the subthreshold beta-related oscillations in membrane potential of corticospinal neurons are more important in determining whether discharge threshold is reached. In summary, convergence at the level of the alpha motor neuron layer allows inputs occurring at the preferred phase of the upstream cortical layer to be conferred a significant timing advantage that is fed forwards. Whether this is a general principle remains to be seen.

The above schema may also help to explain the phase-dependent reduction in the variability of MEP amplitudes. Ordinarily, MEP amplitude will vary with noise, particularly noise that is correlated, whether at the corticospinal or alpha motor neuron level. However, the intrinsic beta rhythm serves to periodically make the corticospinal neuron, and thence the alpha motor neuron, more excitable, so that such noise is no longer needed in combination with the TMS-induced input before neurons can discharge. Our modeling shows that a phase-dependent reduction in the variability of MEP amplitudes can arise through interactions in a very simple circuit, but we should also not discount the possibility that additional dynamics are at play *in vivo* (Womelsdorf et al., 2012).

The difference in mean MEP latency between inputs at the optimal and non-optimal phase of the beta oscillations varied between 0.4 and 0.8 ms, or longer than 1 ms when the very lowest effective stimulation intensities were used. We propose that such small differences in timing may be sufficient to confer advantage at subsequent synapses, where earlier inputs trigger voltage-gated sodium channel-dependent fast action potentials. The all-or-nothing nature of these action potentials, and the refractory period that follows them, effectively may lock out other less optimally timed inputs. The locking-out of less optimally timed inputs might be further promoted by synaptic inhibition, either inhibition by local interneurons that are triggered by the volley of incoming excitation, or feedforward inhibition. However, for any locking-out of less optimally timed inputs to happen small latency advantages need to be retained during propagation. This was the case in the present circuit as the shorter latency of the response to inputs at optimal phase occurred despite the presence of appreciable corticospinal conduction distances, of the intervening synapses with alpha motor neurones and of the membrane time constant of these interposed neurons. A shortening of the latency of the response to inputs at optimal phase might also be important in promoting the strengthening of subsequent synaptic relays through spike-timing-dependent plasticity, if inputs at optimal phase are repeated, and potentiation has been reported under these circumstances (Zanos et al., 2018; Zrenner et al., 2018). Functional impacts of latency differences of the order of 1 ms have also been demonstrated *in vivo* (Tang et al., 2014; Srivastava et al., 2017), and may help explain some of the smaller increases in oscillation frequency reported as a function of stimulus properties, attention or movement (Foffani et al., 2005; van Pelt and Fries, 2013). For example, a 0.5 ms shortening of a cycle of 70 Hz gamma activity would lead to a 2.5 Hz increment in frequency, similar to that demonstrated with attention to a visual stimulus (Bosman et al., 2012). Phase-dependent changes in reaction time have also been reported with visually evoked gamma, although here the latency differences are an order of magnitude greater, and might relate more to the improved transmission gain so that decision thresholds are reached more quickly (Ni et al., 2016).

Regardless of the mechanism by which MEP latency was shortened at optimal phases of cortical beta activity, the faithful propagation of such a small latency advantage means that there is potential for it to be functionally relevant as signals are further transmitted in neural circuits. All told, our data show that the motor system is dynamic even at rest, and that inputs that coincide with windows of raised excitability benefit from more faithful, stronger and rapid transmission to postsynaptic targets.
